# Perilipin 5 alleviates ferroptosis of cardiomyocytes by targeting USP10-p53-TfR proteasome-dependent degradation

**DOI:** 10.3389/fmed.2025.1573230

**Published:** 2025-07-01

**Authors:** Jianqiao Zhao, Jiacheng Shui, Qiyao Xu, Xindong Wang, Yuehong Shen, Chengyuan Liu, Jianping Shen

**Affiliations:** ^1^Department of Cardiology, Affiliated Hospital of Integrated Traditional Chinese and Western Medicine, Nanjing University of Chinese Medicine, Nanjing, Jiangsu, China; ^2^China Academy of Chinese Medical Sciences, Beijing, China; ^3^School of Integrated Chinese and Western Medicine, Nanjing University of Chinese Medicine, Nanjing, China; ^4^Eye Hospital of China Academy of Chinese Medical Sciences, Beijing, China

**Keywords:** PLIN5, ferroptosis, TfR, iron accumulation, myocardial infarction

## Abstract

**Introduction:**

Perilipin 5 (PLIN5) is a key protein attached to lipid droplets that plays a critical role in cellular lipid metabolism. However, its involvement in cardiomyocyte ferroptosis has not been fully elucidated. This study explored the impact of PLIN5 on ferroptosis in H9c2 cells and a rat model of myocardial infarction (MI).

**Methods:**

H9c2 cells were treated with H_2_O_2_ and Erastin, while the rat model of MI was established by ligating the left anterior descending coronary artery.

**Results:**

We found that after MI, cardiac subcellular iron levels increased and the expression of PLIN5 decreased. Overexpression of PLIN5 reduced lipid peroxidation, enhanced ferroptosis resistance, decreased iron accumulation, and lowered TfR expression. Additionally, there was an interaction between PLIN5 and ubiquitin-specific peptidase 10 (USP10). PLIN5 increased the ubiquitination of p53. USP10 and MG-132 blocked the regulatory effect of PLIN5 on TfR expression. Overexpression of USP10 weakened the inhibitory effect of PLIN5 on ferroptosis. *In vivo* experiments showed that overexpression of PLIN5 significantly reduced ferroptosis in the infarcted myocardium.

**Discussion:**

Perilipin 5 may exert cardioprotective effects by regulating the USP10 and p53-TfR axis.

## Introduction

Myocardial infarction (MI) is a cardiovascular condition that poses a significant threat to human health, being a primary contributor to sudden death and heart failure. Due to the interruption of local blood supply to the heart leading to myocardial cell damage and death, patients often have a poor prognosis (1). Studies have shown that the in-hospital mortality rate of ST-segment elevation MI (STEMI) patients is 4.6%–7.5%, with no downward trend in recent years (2). Opening the occluded coronary artery early can preserve viable myocardium or reduce the size of MI. However, early reperfusion is often accompanied by decreased myocardial contractile and diastolic function, reperfusion arrhythmias, changes in myocardial energy metabolism, and alterations in myocardial structure (3). Therefore, there is an urgent need to develop new and more effective drugs for treating acute MI (AMI). Recent studies indicate that during MI, cardiomyocytes can undergo ferroptosis, which may serve as a potential therapeutic target (4). Thus, focusing on the molecular mechanisms of ferroptosis is helpful for developing such drugs.

Understanding the pathogenesis of MI helps in seeking new interventions to reduce myocardial ischemic injury and improve prognosis (5). When MI occurs, cardiomyocytes undergo irreversible damage and necrosis due to reduced oxygen and ATP supply (6). Necrotic cells release their contents, activating the immune system and triggering a severe inflammatory response. Sustained inflammation can cause matrix degradation and cardiomyocyte apoptosis. Excessive reactive oxygen species (ROS) damage cell structures and can lead to cell death, resulting in MI sequelae (7). Ferroptosis is an oxidative stress-related cell death pathway and plays an important role in regulating oxidative stress and inflammatory responses (8, 9). Excessive iron deposition generates oxygen free radicals via the Fenton reaction (10). These radicals react with polyunsaturated fatty acids, causing lipid peroxidation and ultimately destroying phospholipid bilayers (including mitochondrial and plasma membranes) (11). The transferrin receptor (TfR) on the cell membrane internalizes ferrous ions through endocytosis (12). TfR expression levels are considered specific markers of ferroptosis (13). TfR expression can be regulated in multiple ways: (1) transcriptionally by iron-responsive element-binding proteins (IREB) 1/2 (14); (2) post-translationally through ubiquitination, leading to lysosomal degradation (15); and (3) by the transcription factor p53 (16).

Perilipin 5 (PLIN5) is a lipid droplet-associated protein primarily expressed in the liver and muscle tissues. It plays a crucial role in lipid metabolism and lipid droplet dynamics, including regulating fatty acid oxidation, lipid droplet formation and breakdown, and maintaining lipid droplet stability during metabolic stress (17, 18). Studies show that cardiomyocyte-specific knockout of PLIN5 significantly increases ROS levels in MI mouse myocardium (19). Our group’s preliminary work revealed excessive iron accumulation and high lipid peroxidation in ischemic myocardium (20). Given that PLIN5 regulates lipid metabolism and ferroptosis is characterized by lipid peroxidation, the role of PLIN5 in cardiac ferroptosis warrants exploration. Considering the pathophysiology of MI–where lipid metabolism, iron homeostasis, and ROS are intertwined–PLIN5’s physiological functions suggest it may modulate iron-induced cardiomyocyte ferroptosis, providing a novel therapeutic target.

## Materials and methods

### Animals and experimental protocols

All animal experiments followed National Institutes of Health (NIH) guidelines and were approved by the Ethics Committee of Jiangsu Province Academia (no. AEWC-20240120-366). Twenty-four male Sprague-Dawley rats were divided into sham-operated, 3-day, 7-day, and 14-day MI groups. Rats weighed 250 ± 20 g and were fasted for 12 h pre-surgery with free water access. Anesthesia was induced via intraperitoneal injection of 1% pentobarbital sodium (30 mg/kg). After intubation and ventilator connection, rats were positioned supine, and the chest was shaved and disinfected. An incision was made between the 4th and 5th ribs; muscles and pericardium were separated to expose the heart. The left anterior descending coronary artery was ligated below the pulmonary artery using 5–0 sutures. The chest cavity was closed rapidly, air was expelled, and the wound was sutured. Postoperatively, rats recovered on a heating pad. Penicillin (100,000 U) was administered intramuscularly daily for 2 days post-surgery. Sham-operated rats underwent identical procedures without ligation. At designated time points, blood, and heart tissues were collected for histology, iron content measurement, and PLIN5 analysis. Heart tissues were collected from the apical region, including the infarct area and adjacent peri-infarct myocardium (within 2 mm of the infarct border). Distal atrial tissues were not included in this study, as the primary focus was on ischemic cardiomyocytes in the left ventricle. Additionally, 12 Sprague-Dawley rats were divided into AAV9-cTnT-Scramble MI (vector control) and AAV9-cTNT-PLIN5 MI (PLIN5 overexpression) groups. Adenoviruses were injected via the tail vein 3 weeks pre-surgery. Echocardiography was performed on day 6 post-MI; tissues were collected on day 7 for morphology, iron content, and ferroptosis protein analysis.

### Cell culture

H9c2 cells (Servicebio) were maintained in DMEM/F12 medium (Sigma, United States) with 10% fetal bovine serum (Yeason, China) at 37°C/5% CO_2_. Flag-Plin5 and HA-USP10 plasmids (Hanbio) were transfected using Lipofectamine 3000 (Invitrogen, United States). After 24 h transfection, cells were treated with Erastin (10 μM, 24 h) or H_2_O_2_ (400 μM, 2 h). For proteasome inhibition, cells were pre-incubated with 20 μM MG-132 for 24 h. For ubiquitination assays, cells were incubated with 25 μM MG-132 for 8 h before collection.

### Quantitative real-time polymerase chain reaction

Total RNA was extracted using a kit (Vazyme, China) and reverse-transcribed. qPCR was performed using a StepOnePlus system with specific primers (Table 1). Gene expression fold-changes were calculated via the 2^ΔΔCt^ method. Samples were run in duplicate with strict quality control.

**TABLE 1:** The sequence of primers used in real time polymerase chain reaction.

Names	Sequences
F-PLIN5	GTGCCTACAACTCAGCCAAG
R-PLIN5	AGGGCAGCTTCTCTTCCAAT
F- -TfR	CGGCTACCTGGGCTATTGTA
R-TfR	TTCTGACTTGTCCGCCTCTT
F-p53	CATCGAGCTCCCTCTGAGTC
R-p53	GCTTCCTCTGGGCCTTCTAA
F-GAGDH	AAGATGGTGAAGGTCGGTGT
R-GAPDH	AGCTTCCCATTCTCAGCCTT

### Western blotting analysis

Cells were lysed in RIPA buffer (KeyGene, China) with PMSF (1:99). Lysates were centrifuged at 12,000 × *g* for 20 min. Protein concentration was determined using a BCA kit (Beyotime, China). Proteins were separated by SDS-PAGE, transferred to PVDF membranes, and blocked with 5% skim milk for 1 h. Membranes were incubated with primary antibodies overnight at 4°C, followed by Horseradish peroxidase (HRP)-conjugated secondary antibodies for 1 h. Signals were detected using chemiluminescence (Tanon 5200, China). Antibodies: TfR (1:200, Santa Cruz), ACSL4 (1:1,000, Abcam), GPX4 (1:1,000, Abmart), PLIN5 (1:1,500, Abclonal), USP10 (1:1,000, Proteintech), and GAPDH (1:5,000, FdBio).

### Iron assay

Rat myocardial tissue (100 mg) was homogenized in lysis buffer. Ferrous ion content was measured per kit instructions. After chromogen addition, samples were incubated at 37°C for 30 min, and absorbance (590 nm) was read. For cellular iron, FerroOrange (Dojindo, Japan) was used. All fluorescence images were captured using a fixed exposure time (100 ms) and identical magnification (400×) across experimental groups. Background subtraction was performed using ImageJ’s rolling ball algorithm (radius = 30 pixels) to remove non-specific signal uniformly without altering cellular fluorescence intensity. Cells were washed with phosphate-buffered saline (PBS), incubated with 1 μM probe for 30 min, and imaged (ex: 543 nm, em: 580 nm).

### BODIPY staining

Cells were incubated with 10 μM BODIPY 581/591 (in DMSO) for 30 min, washed with PBS, and imaged. Oxidized lipids (green) and non-oxidized lipids (red) were quantified using ImageJ; the green/red ratio indicated lipid peroxidation. BODIPY 581/591 staining was performed as described, with all samples processed and imaged simultaneously to ensure consistency. The increased green fluorescence in Erastin-treated cells reflects elevated lipid peroxidation, a known hallmark of ferroptosis.

### Immunoprecipitation and mass spectrometry

Protein samples were immunoprecipitated using anti-FLAG magnetic beads ± Erastin. SDS-PAGE gels were stained with Coomassie Brilliant Blue (CBB). Bands were analyzed by LC-MS and matched to the National Center for Biotechnology Data protein database.

### Co-immunoprecipitation

For immunoprecipitation/co-immunoprecipitation (Co-IP) experiments, H9c2 cells were transfected with Flag-PLIN5 plasmids for 24 h, followed by treatment with Erastin (10 μM) for an additional 24 h. Cell lysates were prepared for immunoprecipitation using anti-FLAG or anti-USP10 antibodies. Rinse cells and lyse with cold PBS. Perform Co-IP on cell lysates using an IP/Co-IP kit (BersinBio, China). Lyse all samples with the kit-provided IP lysis buffer. Incubate Protein A/G magnetic beads with lysates (50% volume) at 4°C for 2 h. After overnight incubation with primary antibodies at 4°C, wash beads three times with lysis buffer. Resuspend immunoprecipitated proteins in loading buffer and boil at 95–100°C for 5 min. Separate proteins by SDS-PAGE. For target protein pulldown, perform immunoprecipitation using p53 (CST, United States) and FLAG (Abclonal, China) antibodies. Finally, detect USP10 (Proteintech, China) and ubiquitin (Ub) (Santa Cruz, United States) by immunoblotting.

### Immunohistochemistry

Heart tissues were embedded in paraffin, sectioned, deparaffinized in xylene, and rehydrated through graded ethanol solutions. Tissue sections were then incubated with PLIN5 primary antibody (1:200, Abclonal, China) for 2 h at room temperature. After washing three times with PBS, sections were sequentially incubated with: (1) HRP-conjugated secondary antibody (ServiceBio, China) for 1 h; (2) 3,3′-Diaminobenzidine (DAB) solution (ServiceBio, China) for color development. Protein expression was assessed by quantifying brown staining intensity. Immunohistochemistry was performed using a standard DAB protocol. Due to constraints in fluorescent microscopy availability, results are presented as brightfield images. PLIN5 expression was validated via Western blot and quantitative real-time polymerase chain reaction (qRT-PCR). 4-HNE staining for lipid peroxidation was performed using a validated antibody (1:200, Abcam) and imaged at 200× magnification. Due to variability in tissue processing, signals were quantified by integrated optical density (IOD) rather than qualitative assessment.

### GSEA analysis

Gene expression data from PLIN5-overexpressed myocardial tissue and wild-type controls were retrieved from the GEO dataset GSE44192. Ferroptosis-related gene sets (WP_FERROPTOSIS), intracellular iron homeostasis (GOBP_INTRACELLULAR_IRON_ION_HOMEOSTASIS), and iron transport (GOBP_IRON_ION_TRANSPORT) were downloaded from the MsigDB database. Input files were formatted according to GSEA v4.0.1 specifications to analyze the differences in gene expression between groups and the enrichment of gene sets related to intracellular iron homeostasis, cellular iron uptake and transport, and ferroptosis.

### Statistical analysis

All quantitative data are presented as mean ± standard deviation (SD). The Shapiro–Wilk test assessed normality, while the *F*-test evaluated homogeneity of variances. When data met both normality and equal variance assumptions, group comparisons used Student’s *t*-test. For data violating either assumption, Welch’s corrected *t*-test was applied. Multiple group comparisons with normally distributed data employed one-way analysis of variance (ANOVA), followed by Tukey’s post-hoc test for inter-group comparisons. Statistical significance was defined as *P* < 0.05.

## Results

### PLIN5 is downregulated in MI

**FIGURE 1:**
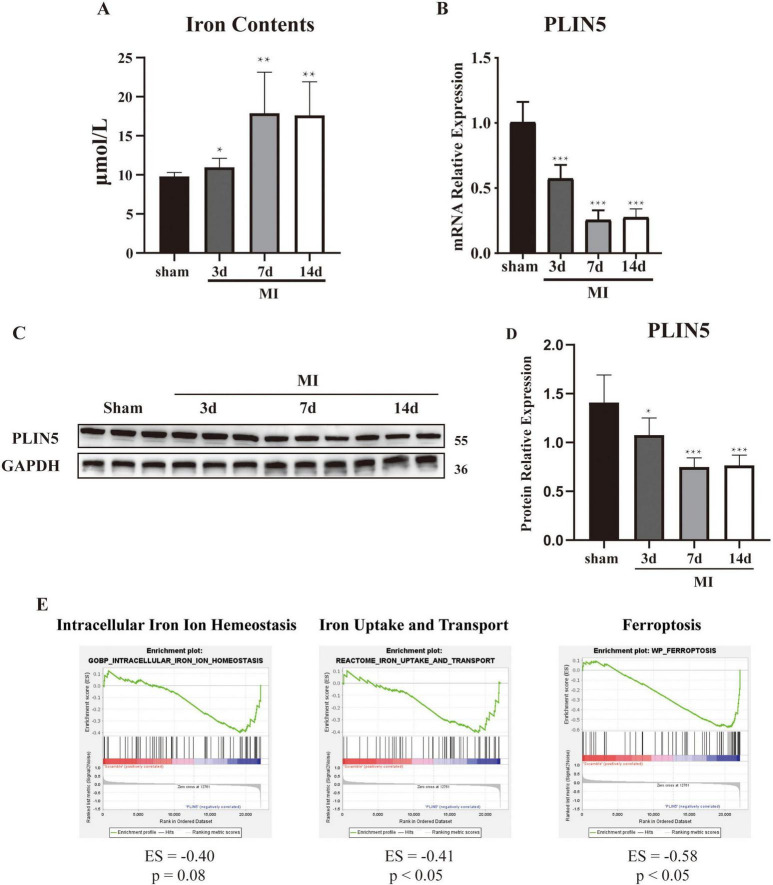
Perilipin 5 (PLIN5) is downregulated in myocardial infarction. **(A)** Ferrous ion concentration in heart tissue. **(B)** mRNA expression level of PLIN5 in the anterior infarct heart tissue of sham and myocardial infarction rats. **(C,D)** Protein expression of PLIN5 in heart tissue of sham and myocardial infarction rats by Western blot. **(E)** GSEA analysis of GSE44192 dataset. All data are presented as mean ± SD (*n* = 6). *P < 0.05, **P < 0.01, ***P < 0.001, vs. sham. Scale bar, 100 μm.

**FIGURE 2:**
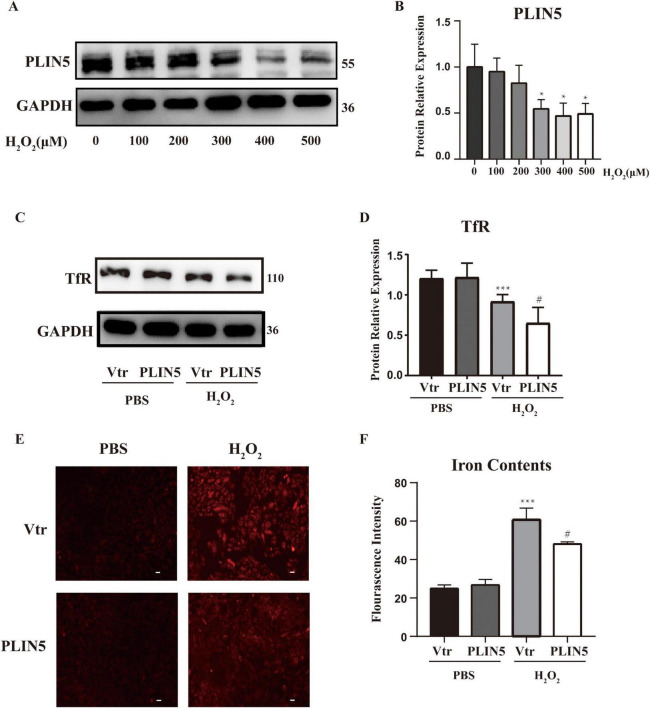
Perilipin 5 (PLIN5) attenuates iron accumulation in H_2_O_2_ treated cardiac cells. **(A,B)** PLIN5 protein expression in H9c2 cells was assessed via Western blotting after exposure to varying concentrations of H_2_O_2_ for 2 h. *P < 0.05, vs. 0 μM. **(C,D)** Protein levels of TfR in H9c2 cells by exposure to H_2_O_2_ (400 μM, 2 h) transfected with PLIN5 plasmids or its control vectors by immunoblotting. ***P < 0.001 vs. PBS + Vtr; ^#^*P* < 0.05 vs. H_2_O_2_ + Vtr. **(E,F)** Effect of PLIN5 on iron contents by FerroOrange staining in H9c2 cells treated with H_2_O_2_ (400 μM, 2 h). ***P < 0.001 vs. PBS + Vtr; ^#^*P* < 0.05 vs. H_2_O_2_ + Vtr. All data were presented as mean ± SD (*n* = 3).

To characterize temporal changes in myocardial ferrous iron levels post-MI, we quantified ferrous ion content at 3, 7, and 14 days post-surgery vs. sham controls. MI significantly increased myocardial ferrous iron at all timepoints (Figure 1A). Parallel dynamic assessment revealed progressive PLIN5 downregulation: (1) mRNA decreased significantly at 3/7/14 days post-MI (Figure 1B); (2) protein expression declined correspondingly (Figures 1C, D); and (3) immunohistochemistry confirmed reduced myocardial PLIN5 (Supplementary Figure 1). To establish ferrous iron-PLIN5 linkage, we analyzed GEO dataset GSE44192. GSEA demonstrated significant enrichment of ferroptosis-related genes and iron transport genes in PLIN5 expression vs. wild-type cardiomyocytes. No enrichment occurred for intracellular iron homeostasis genes (Figure 1E). These pathways are biologically relevant to human MI, as iron-dependent lipid peroxidation is a conserved feature of ischemic cardiomyopathy.

## PLIN5 attenuates iron accumulation in H_2_O_2_ treated cardiac cells

To model the post-MI oxidative microenvironment, we induced oxidative stress in cardiomyocytes using H_2_O_2_ and examined PLIN5’s impact on intracellular ferrous iron levels. H_2_O_2_ treatment (2 h) dose-dependently reduced PLIN5 protein expression at 300, 400, and 500 μM vs. controls (Figures 2A, B). To elucidate PLIN5’s role in ferrous iron regulation under oxidative stress, we overexpressed PLIN5 (transfection efficiency validated by qPCR; Supplementary Figure 2). Analysis of transferrin receptor 1 (TfR), a critical iron uptake transmembrane protein, revealed that H_2_O_2_ (400 μM, 2 h) significantly decreased TfR expression. PLIN5 overexpression further reduced TfR protein levels (Figures 2C, D). This H_2_O_2_-induced TfR downregulation likely represents a cellular feedback mechanism to prevent iron overload. Parallel FerroOrange assays demonstrated elevated intracellular ferrous iron accumulation in H_2_O_2_-treated H9c2 cells vs. controls. PLIN5 overexpression significantly attenuated this ferrous iron increase (Figures 2E, F).

### The inhibitory effect of PLIN5 on erastin-induced ferroptosis

Based on the study results showing that PLIN5 reduces intracellular ferrous ion content and downregulates TfR expression under oxidative stress, we hypothesized that PLIN5 is involved in cardiomyocyte iron metabolism and may impact ferroptosis. Therefore, we used Erastin to trigger ferroptosis in cardiomyocytes. First, we observed that Erastin (10 μM, 24 h) stimulation led to an increase in the expression of Acyl-CoA Synthetase Long-Chain Family Member 4 (ACSL4). However, application of the iron chelator deferoxamine (DFO) reversed this increase in ACSL4 expression (Supplementary Figure 3).

**FIGURE 3:**
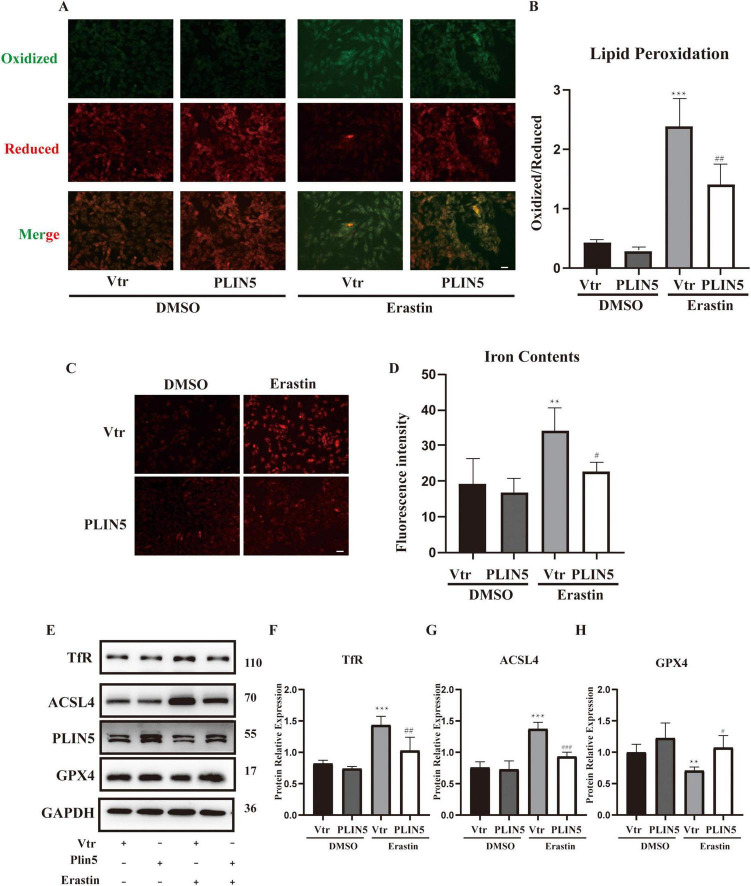
Perilipin 5 (PLIN5) confers resistance to erastin-induced ferroptosis in H9c2 cells. **(A,B)** BODIPY staining of H9c2 cells treated by Erastin with or without transfection of PLIN5 (*n* = 4). **(C,D)** Intracellular iron contents of H9c2 cells treated by Erastin with or without transfection of PLIN5 and USP10 by FerroOrange staining (*n* = 4). **(E–H)** Protein expression of TfR, ACSL4, and GPX4 by WB (*n* = 3). **P < 0.01, ***P < 0.001 vs. Vtr + DMSO; ^#^*P* < 0.05, ^##^*P* < 0.05, ^###^*P* < 0.001 vs. Vtr + Erastin.

We transfected PLIN5 into cardiomyocytes and observed its effect on Erastin-induced ferroptosis. Using BODIPY staining to assess ferroptosis-associated lipid peroxidation levels, we found that Erastin significantly increased lipid peroxidation in cardiomyocytes. However, PLIN5 overexpression significantly reduced these levels (Figures 3A, B). Measurement of intracellular ferrous iron levels with the FerroOrange probe also revealed that Erastin significantly increased ferrous iron compared to the control group, while PLIN5 overexpression significantly reduced intracellular ferrous iron accumulation (Figures 3C, D).

Acyl-CoA Synthetase Long-Chain Family Member 4 plays a crucial role in ferroptosis by converting free long-chain fatty acids into fatty acyl-CoA esters. GPX4, as an antioxidant enzyme, reduces lipid peroxides during ferroptosis. Evaluation of ACSL4 and GPX4 levels showed that Erastin treatment significantly upregulated ACSL4 and downregulated GPX4. PLIN5 overexpression markedly reversed these protein expression changes (Figures 3E–H).

Given PLIN5’s potential role in iron metabolism, we investigated its impact on key iron metabolism-related proteins under ferroptosis induction, including Iron Responsive Element Binding Proteins (IREB1 and IREB2), ferritin heavy chain (FTH), TfR, and ferroportin (FPN). Erastin stimulation significantly increased FTH and TfR expression while significantly decreasing FPN expression, with no significant effect on IREB1 or IREB2. PLIN5 overexpression in cardiomyocytes significantly decreased only TfR expression, without significantly affecting FPN or FTH levels (Supplementary Figure 4).

### PLIN5 regulates TfR in a proteasome-dependent mannar

**FIGURE 4:**
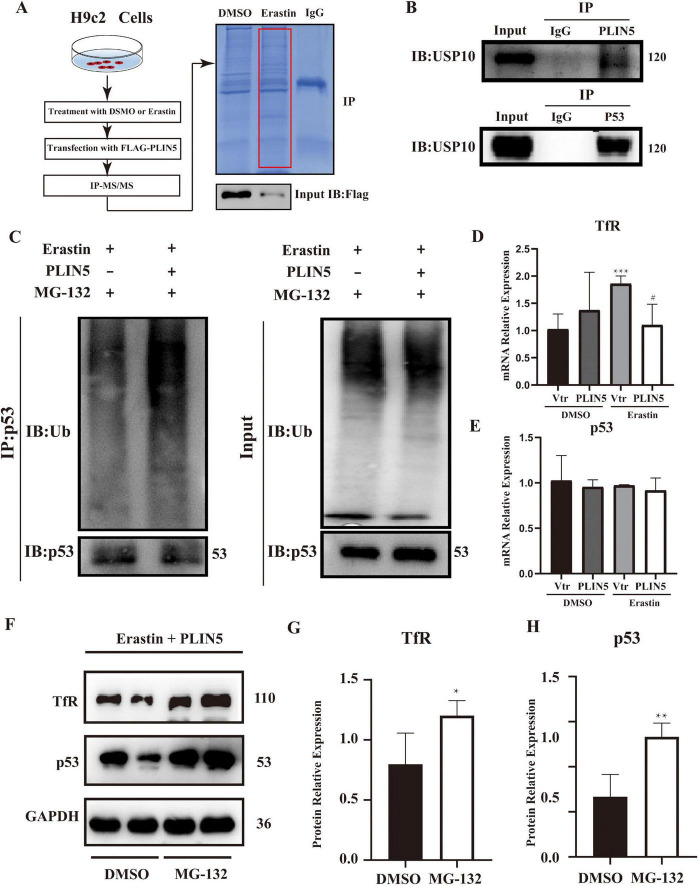
Perilipin 5 (PLIN5) regulates TfR in a proteasome-dependent mannar. **(A)** Overexpression of FLAG-tagged PLIN5 in H9c2 cardiomyocytes. After transfection, FLAG antibody was used for immunoprecipitation (IP), followed by protein gel electrophoresis and Coomassie Brilliant Blue staining. **(B)** Co-immunoprecipitation (Co-IP) assay to detect interactions between PLIN5 and USP10, p53 proteins. **(C)** Impact of PLIN5 on p53 ubiquitination levels under proteasome inhibitor MG-132 treatment. **(D,E)** qPCR analysis of TfR and p53 mRNA expression in H9c2 cells post-Erastin treatment. ***P < 0.001 vs. Vtr + PBS; ^#^*P* < 0.05 vs. Vtr + Erastin. **(F–H)** Western blot analysis exploring the effect of MG-132 on TfR and p53 protein expression. *P < 0.05, **P < 0.01 vs. DMSO; *n* = 4.

To further investigate how PLIN5 regulates iron metabolism-related genes, considering its effect on TfR, we employed Immunoprecipitation-Mass Spectrometry (IP-MS) to identify PLIN5-binding proteins. Database analysis revealed potential interactions between PLIN5 and USP10, p53, and TfR (Figure 4A). Subsequent Co-IP confirmed that PLIN5 interacts with USP10, and that USP10 binds p53 (Figure 4B). Notably, PLIN5 and p53 did not co-immunoprecipitate under tested conditions, despite both binding to USP10. This suggests a competition model where PLIN5 sequesters USP10, preventing its deubiquitination of p53. Moreover, while PLIN5 does not directly interact with TfR, its overexpression reduces p53 protein levels, a known transcription factor for TfR. This suggests an indirect role for PLIN5 in TfR downregulation via p53 degradation. To determine whether PLIN5 regulates TfR through the USP10-p53-TfR axis, we first examined PLIN5’s impact on p53 ubiquitination. During Erastin-induced ferroptosis, proteasome inhibitor MG-132 (25 μM, 8 h) blocked proteasomal degradation, while PLIN5 overexpression enhanced p53 ubiquitination (Figure 4C). PLIN5 significantly reduced TfR mRNA expression but did not affect p53 mRNA levels in Erastin-treated cardiomyocytes (Figures 4D, E). When proteasomal degradation was inhibited by MG-132 (20 μM, 24 h), p53 protein accumulation increased, concomitantly elevating TfR protein expression (Figures 4F–H). These results indicate that PLIN5 regulates p53 through post-translational modification by increasing its ubiquitination. This reduces TfR expression, likely due to decreased p53 levels suppressing TfR transcription.

### USP10 mediates PLIN5’s role of resistance to ferroptosis

**FIGURE 5:**
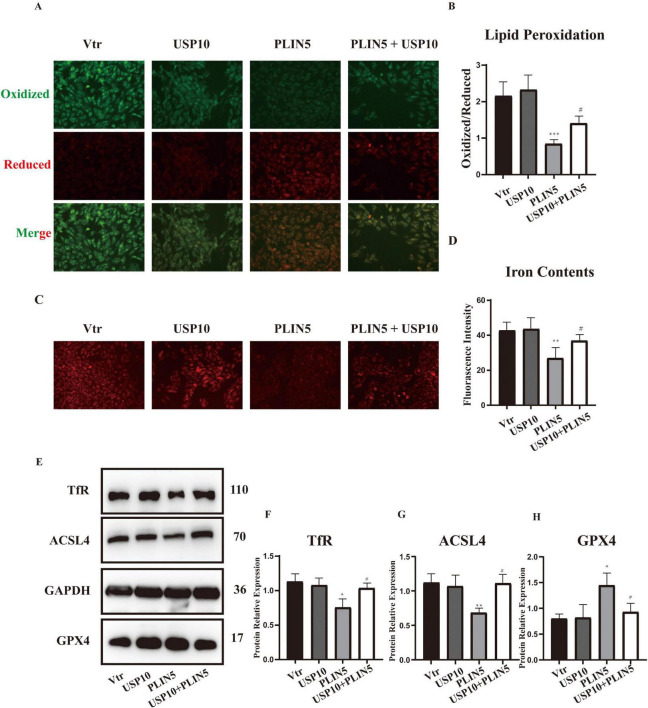
Ubiquitin-specific peptidase 10 (USP10) mediates PLIN5’s role of resistance to ferroptosis. **(A,B)** BODIPY staining of H9c2 cells with or without transfection of PLIN5 and USP10 (*n* = 5). **(C,D)** Intracellular iron contents of H9c2 cells with or without transfection of PLIN5 and USP10 by FerroOrange staining (*n* = 5). **(E–H)** Protein expression of TfR, ACSL4, and GPX4 by WB (*n* = 3). *P < 0.05, **P < 0.01, ***P < 0.001 vs. Vtr; ^#^*P* < 0.05 vs. PLIN5.

To further demonstrate that PLIN5 reduces TfR expression and alleviates ferroptosis through USP10 mediation, we co-transfected cardiomyocytes with USP10 and PLIN5 alongside their respective control plasmids under the context of erastin. Compared to PLIN5 transfection, which reduced lipid peroxidation levels, USP10 transfection significantly increased lipid peroxidation (Figures 5A, B). Erastin treatment did not alter USP10 protein levels, indicating that PLIN5 regulates USP10 activity rather than transcriptional/translational expression. Similarly, FerroOrange staining revealed that while PLIN5 lowered intracellular ferrous iron levels, USP10 overexpression promoted ferrous iron accumulation (Figures 5C, D). USP10 also substantially reversed PLIN5-mediated effects on ACSL4, GPX4, and TfR expression in cardiomyocytes (Figures 5E–H).

### PLIN5 alleviates ferroptosis *in vivo*

**FIGURE 6:**
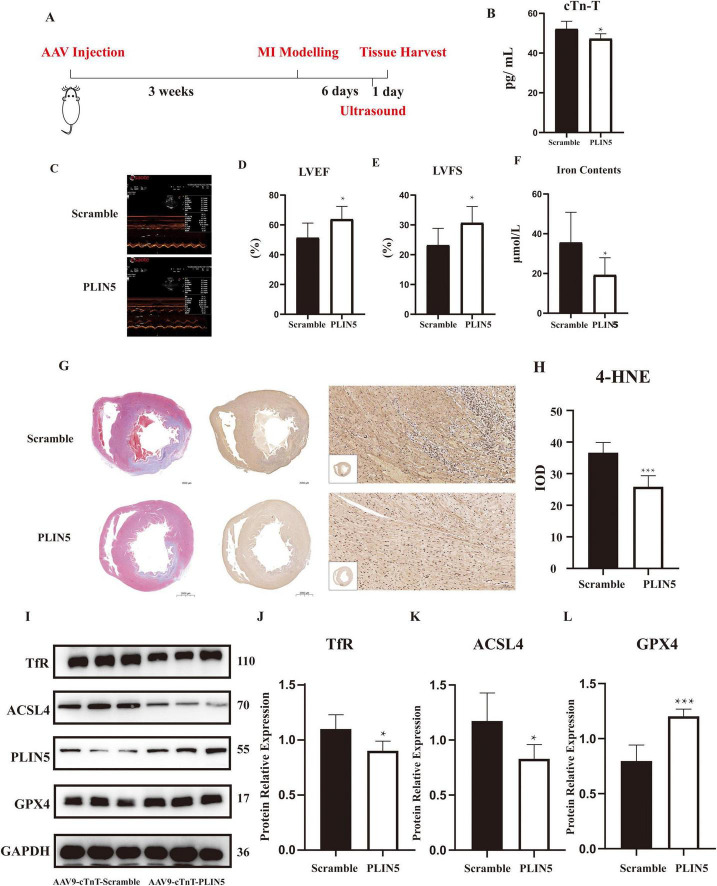
Perilipin 5 (PLIN5) alleviates ferroptosis *in vivo* (*n* = 6). **(A)** The experimental schedule for investigating the effect of overexpression of PLIN5 on ferroptosis in rats. **(B)** Serum levels of cTn-T in rats injected with PLIN5 AAV9 virus or its control (*n* = 6). **(C–E)** The measurement of left ventricular ejection fraction and fractional shortening by Echocardiography (*n* = 6). **(F)** Ferrous ion concentration in rats injected with PLIN5 AAV9 virus or its control (*n* = 6). **(G,H)** Masson’s trichrome staining and 4-HNE immunohistochemistry of rats injected with PLIN5 AAV9 virus or its control (*n* = 6). IOD, integrated optical density. **(I–L)** TfR, ACSL4, and GPX4 protein expression in PLIN5 overexpressed or its control rats by WB experiments (*n* = 6). *p < 0.05, ***p < 0.001 vs. Scramble.

*In vitro* experiments indicate that PLIN5 reduces intracellular ferrous iron accumulation via USP10 to protect against ferroptosis. We further validated these findings by intravenously injecting adenoviruses carrying PLIN5 or control vectors into rat tail veins before MI, assessing their effects on post-MI cardiac function and ferroptosis (Figure 6A). cTn-T concentrations were significantly reduced in PLIN5-overexpressing rats (Figure 6B). Moreover, the PLIN5 overexpression group exhibited significantly increased left ventricular ejection fraction (LVEF) and fractional shortening (LVFS), indicating improved cardiac function (Figures 6C–E). Intracellular ferrous iron levels in myocardial tissues were also significantly lower in this group compared to the AAV-Scramble controls (Figure 6F).

Masson’s trichrome staining revealed substantial MI lesions in the anterior walls of both groups (Figure 6G). While visual assessment of 4-hydroxynonenal (4-HNE) staining is challenging, quantitative IOD analysis showed a 2.1-fold increase in lipid peroxidation in MI hearts vs. sham, which was reduced by 45% in PLIN5-overexpressing hearts (Figure 6H). Western blot analysis of ferroptosis-related proteins showed that PLIN5 overexpression significantly decreased ACSL4 and TfR expression while increasing GPX4 levels compared to AAV-Scramble controls (Figures 6I–L).

## Discussion

This study demonstrates PLIN5’s role in regulating iron metabolism and ferroptosis. PLIN5 alleviates Erastin-induced ferroptosis by reducing excessive intracellular ferrous iron accumulation. We found that PLIN5 decreases cellular iron content by downregulating TfR expression, thereby reducing ferrous iron aggregation—an effect blocked by proteasome inhibitors. Mechanistically, PLIN5 overexpression inhibits the USP10-p53-TfR axis, potentially through competitive binding with USP10. This interaction inhibits USP10-mediated deubiquitination of p53, enhancing p53’s proteasome-dependent degradation and ultimately reducing TfR transcription. USP10 overexpression attenuates PLIN5-mediated TfR downregulation and suppresses PLIN5’s effects on intracellular ferrous iron accumulation and ferroptosis. In human MI patients, iron accumulation in cardiomyocytes correlates with TfR upregulation, while lipid droplet proteins like PLIN2 show reduced expression in ischemic myocardium (21). Although PLIN5-specific human data are pending, our findings align with the broader role of lipid-iron crosstalk in MI pathophysiology.

Iron is an essential component for sustaining the high metabolic demands of the cardiovascular system. However, excessive iron accumulation in cardiac tissue is detrimental, making iron homeostasis crucial for maintaining optimal cardiovascular physiology (22). In post-MI heart failure patients, excess iron accumulates in cardiac mitochondria, exacerbating disease progression. Similarly, iron levels increase during murine myocardial ischemia. This iron overload triggers ROS overproduction, inhibits respiratory chain enzymes, and impairs ATP synthesis (23). Through the Fenton reaction, iron ions generate hydroxyl radicals—the strongest biological oxidants—which damage biomembranes and catalyze phospholipid/polyunsaturated fatty acid peroxidation. Consequently, iron is indispensable for ferroptosis. The TfR, responsible for cellular iron uptake, serves as a key ferroptosis marker.

Perilipin 5 contains domains that interact with adipose triglyceride lipase and hormone-sensitive lipase, promoting triglyceride and diglyceride hydrolysis to facilitate lipid breakdown (24). By modulating lipid droplet storage functions and anchoring droplets to mitochondria, PLIN5 alleviates diabetic cardiomyopathy through PGC1α-PPARα axis activation—a key regulatory pathway in energy metabolism (25). Lipid and iron metabolism are intimately linked: iron supplementation increases fatty acid uptake and low-density lipoprotein content, while fatty acid excess induces iron pool accumulation (26, 27). This study elucidates the lipid-iron metabolism interplay by investigating PLIN5-mediated TfR regulation.

Our immunoprecipitation experiments identified a potential PLIN5-USP10 interaction. USP10—a deubiquitinating enzyme regulating cell growth, apoptosis, and autophagy—modulates TfR expression in conjunction with MG-132. USP10 overexpression attenuates PLIN5’s inhibition of ferroptosis, potentially disrupting cellular iron homeostasis. The E3 ubiquitin ligase mouse double minute 2 homolog (MDM2) governs p53 stability by promoting its ubiquitination and proteasomal degradation. USP10 stabilizes p53 by inhibiting MDM2-mediated ubiquitination (28). *In vivo*, PLIN5 overexpression significantly reduced ferroptosis in infarcted myocardium, while *in vitro* data suggest cardioprotection occurs via USP10-p53-TfR axis regulation. This PLIN5-USP10-p53-TfR interplay may influence cardiac oxidative stress, apoptosis, and iron metabolism. USP10 modulates p53 activity (29), and the p53-TfR axis regulates iron homeostasis under stress (30).

Several mechanisms require clarification: PLIN5’s physiological role in hypoxic H9c2 cells remains undefined, and evidence for PLIN5-mediated ferrous iron reduction in ischemic tissue is limited. While PLIN5 transports monounsaturated fatty acids to activate SIRT1 (31), its potential non-lipid droplet functions warrant exploration. The USP10-PLIN5 binding site requires validation to determine direct/indirect interaction. Our Erastin-induced ferroptosis model differs pathophysiologically from clinical MI, necessitating studies on ferroptosis inhibition during actual ischemic injury.

A limitation of this study is the absence of PLIN5 knockdown experiments, which would further validate its causal role in ferroptosis regulation. However, our gain-of-function data consistently show that PLIN5 overexpression suppresses iron accumulation and lipid peroxidation, while MI-induced PLIN5 downregulation correlates with enhanced ferroptosis. Technical challenges in achieving efficient PLIN5 knockdown in primary cardiomyocytes (e.g., low transfection efficiency with shRNA and off-target effects of CRISPR/Cas9) precluded these experiments during the study period. Future research using conditional PLIN5 knockout mice or improved knockdown tools will be essential to confirm these mechanisms. The absence of human MI tissue validation is another limitation. Future studies using clinical samples or induced pluripotent stem cell (iPSC)-derived cardiomyocytes from MI patients could directly assess PLIN5-TfR co-expression and ferroptosis markers. Until then, our findings in rodent models provide a preclinical foundation for targeting this axis in human disease. Furthermore, a notable limitation is the reliance on Erastin, a glutaminase inhibitor, to induce ferroptosis *in vitro*. While Erastin effectively models acute oxidative stress, it does not fully replicate the multifaceted MI microenvironment, including hypoxia, nutrient deprivation, and reperfusion injury. Future studies should employ alternative ferroptosis inducers like RSL3 or hypoxia/reoxygenation models to validate PLIN5’s protective effects under conditions more reflective of clinical MI. Additionally, direct assessment of ferroptosis markers during *in vivo* ischemia-reperfusion would further solidify the translational relevance of our findings. Moreover, a technical limitation is the modest IHC staining quality, which may reflect antibody sensitivity or tissue preservation. Future studies using fluorescent immunohistochemistry or confocal microscopy will be needed to precisely localize PLIN5 in cardiomyocytes vs. non-myocardial cells. In addition, the absence of PLIN5-p53 Co-IP data is a limitation, the observed competition between PLIN5 and p53 for USP10 binding is inferred from indirect evidence and requires validation via biophysical methods or structural modeling in future studies.

This study revealed that PLIN5, typically associated with lipid metabolism, participates in the physiological processes of iron metabolism and ferroptosis. These results provides evidence of the relationship between lipids and iron, contributing to the development of therapeutic drugs targeting MI.

## Data Availability

The raw data supporting the conclusions of this article will be made available by the authors, without undue reservation.
