# Supplementary material for: Perilipin 5 alleviates ferroptosis of cardiomyocytes by targeting USP10-p53-TfR proteasome-dependent degradation
Source: Front Med (Lausanne). 2025 Jul 1;12:1573230. doi: 10.3389/fmed.2025.1573230 (PMC12259547; doi:10.3389/fmed.2025.1573230)

**Supporting information Figure:**

**
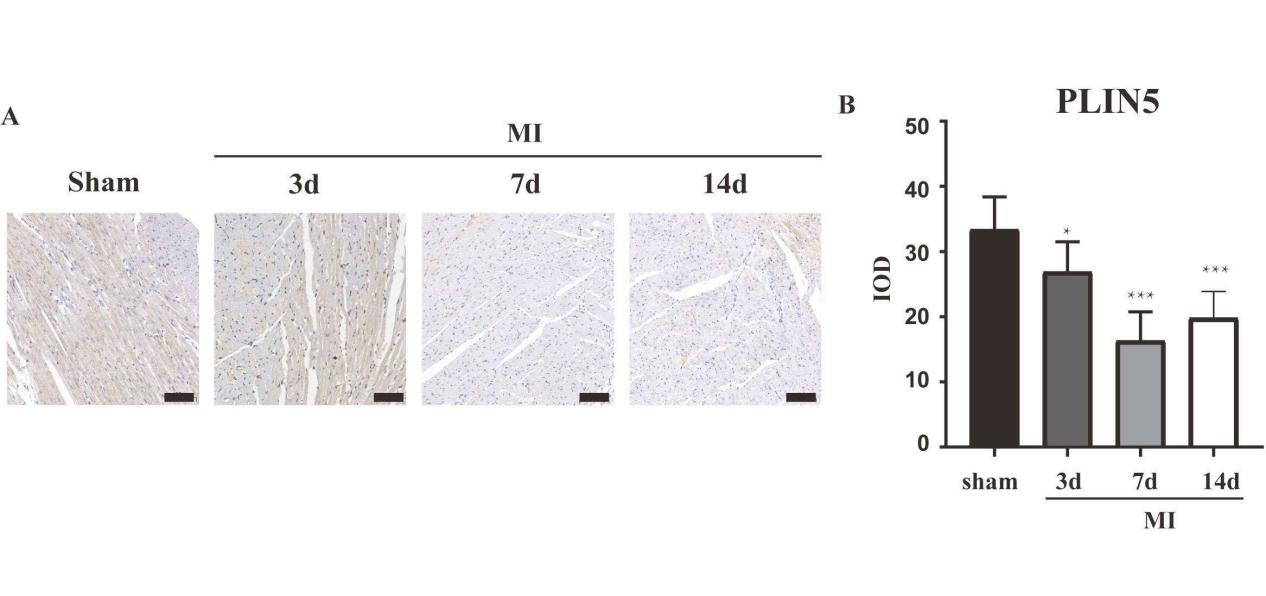
**

Figure S1. Immunohistochemistry for myocardial PLIN5


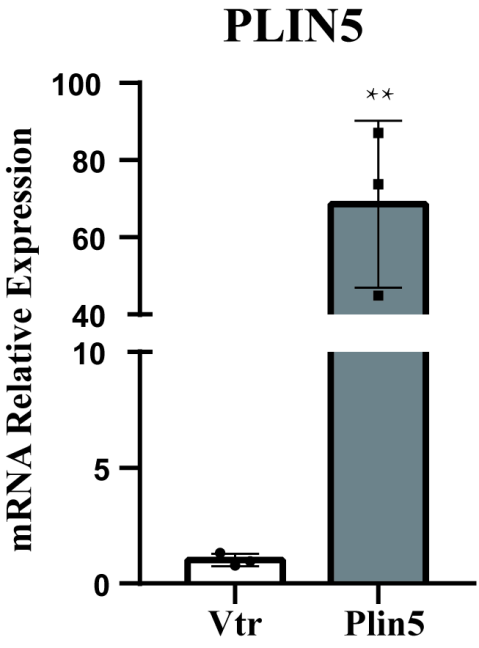


Figure S2. transfection efficiency by transfected PLIN5 plasmids.


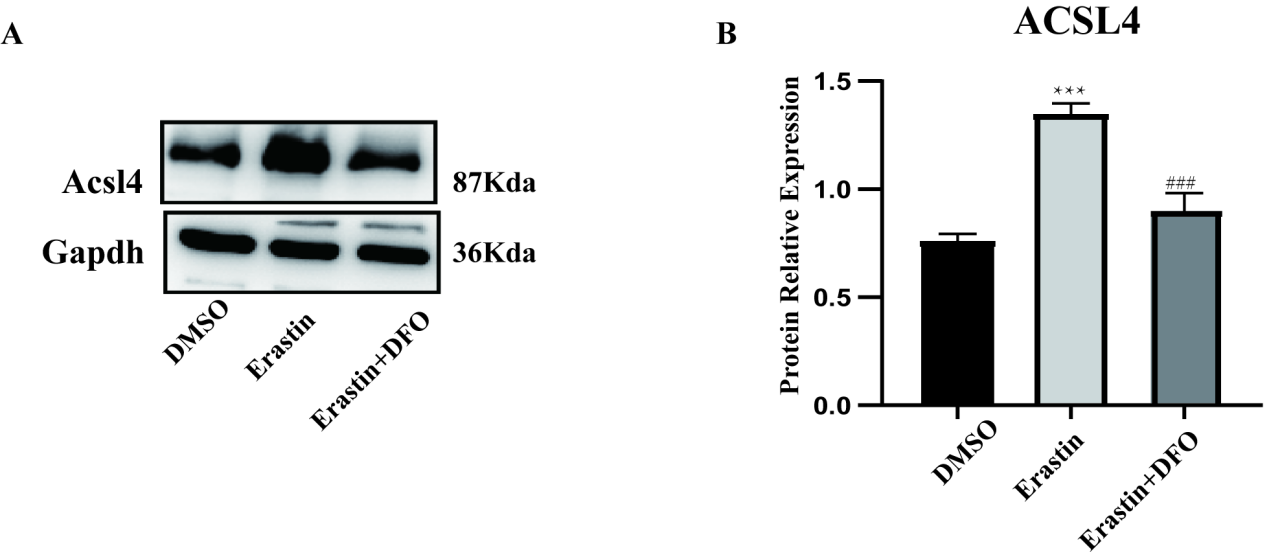


Figure S3. The ACSL4 expression by using the iron chelator deferoxamine.


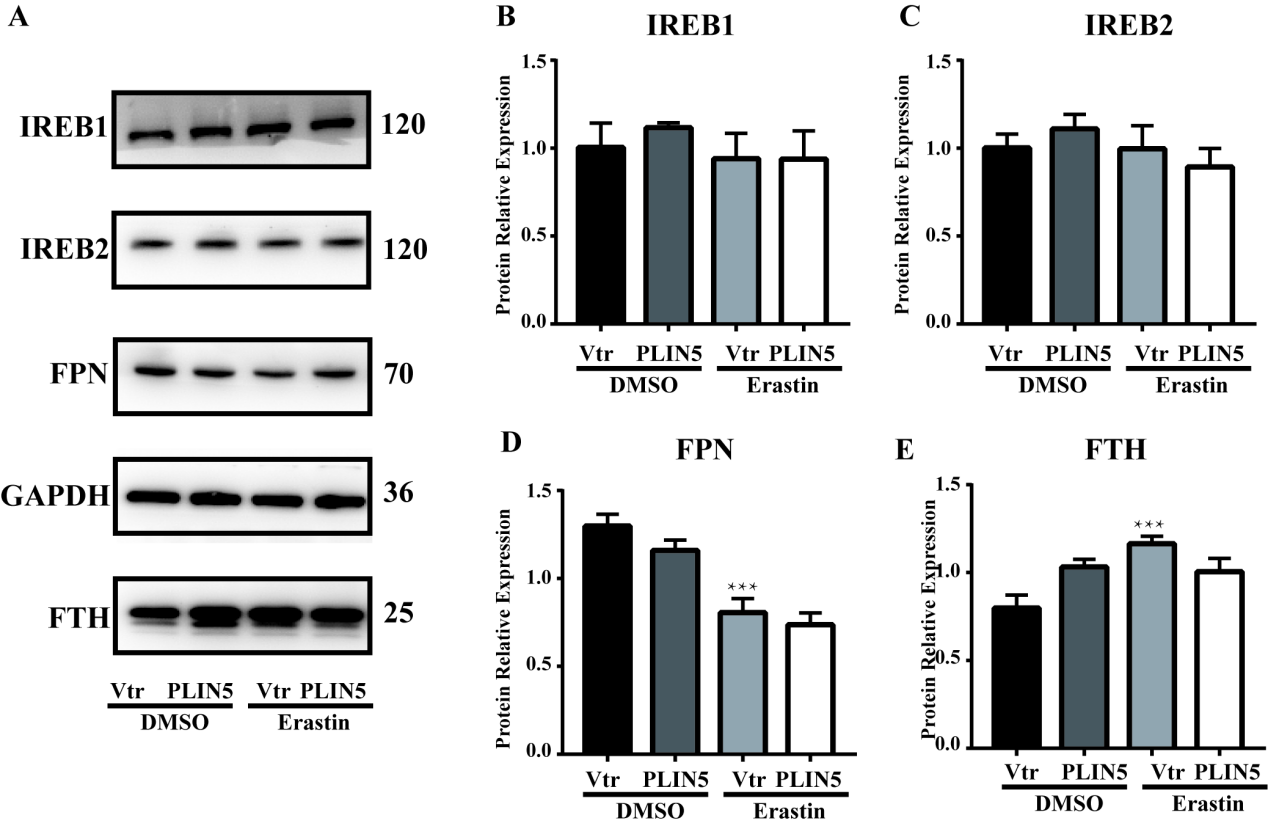


Figure S4. The impact of PLIN5 on the expression of IREB1, IREB2, FTH, and FPN.

ORIGINAL BLOTS AND GELS:

Fig 1 PLIN5


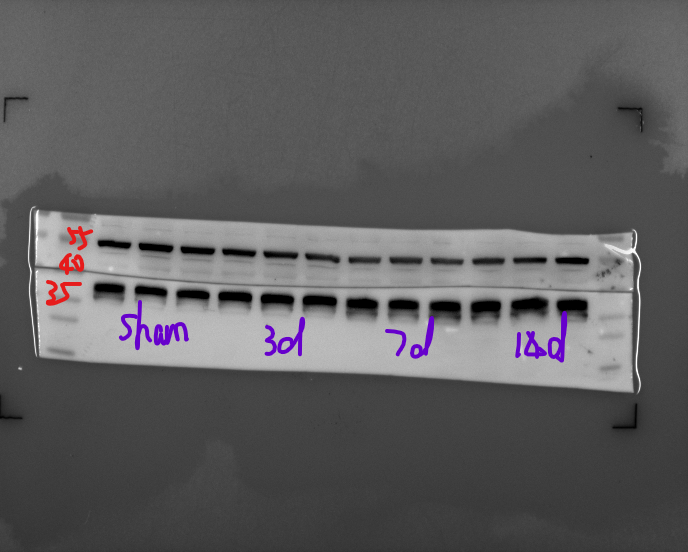


Fig 2 TFRC-gap-2


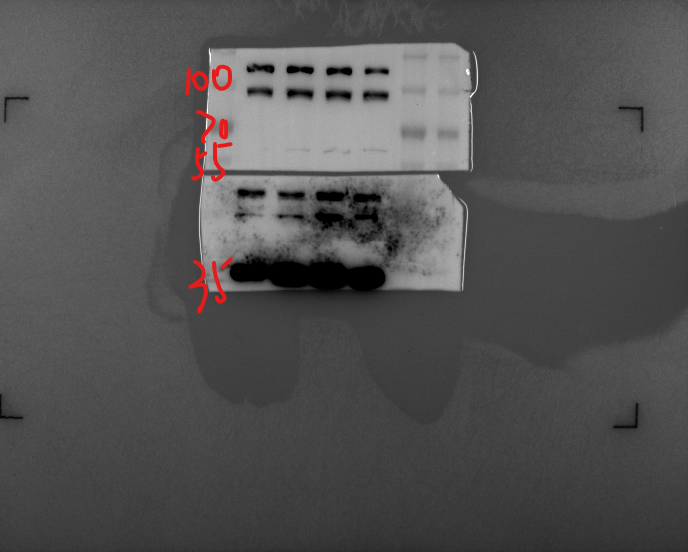


Fig 3 ACSL4


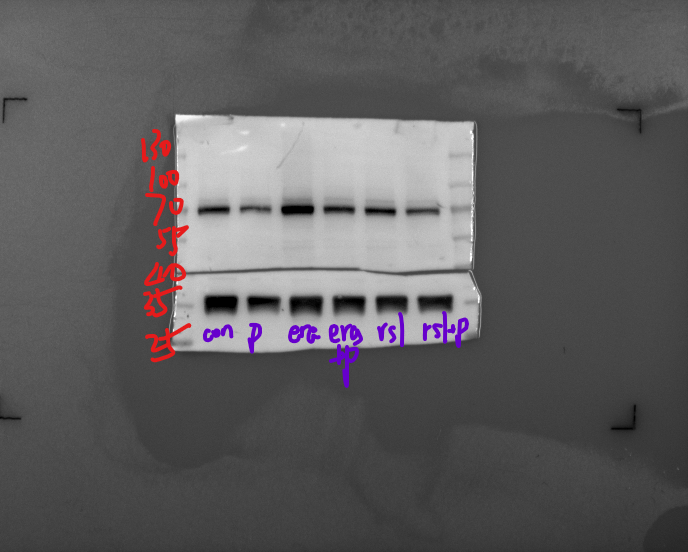


Fig 3 GPX4


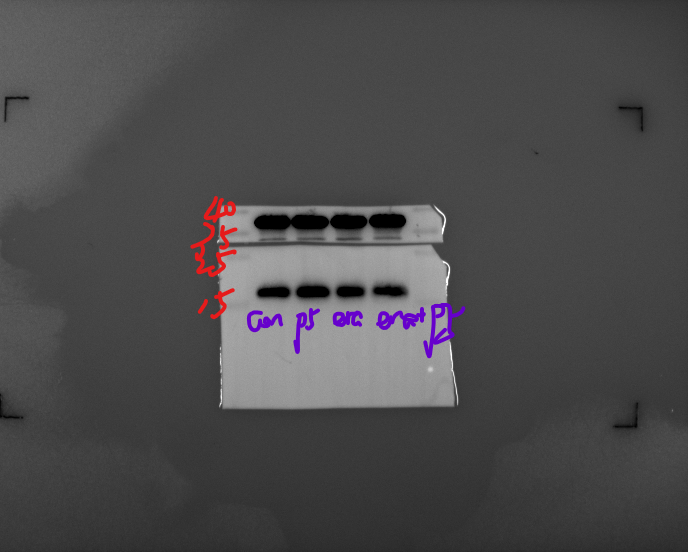


Fig 3 GPX4-2


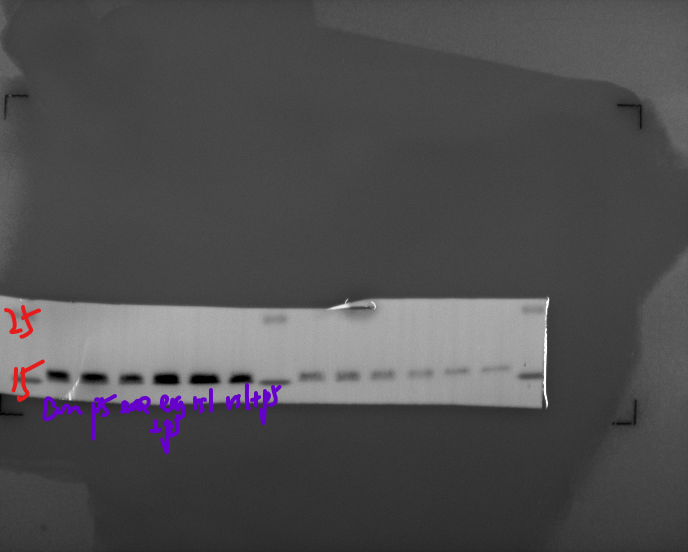


Fig 3 PLIN5


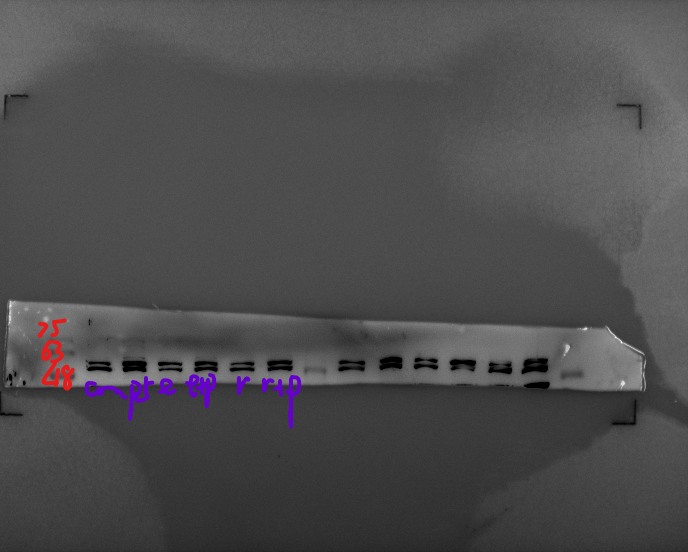


Fig 3 TfR


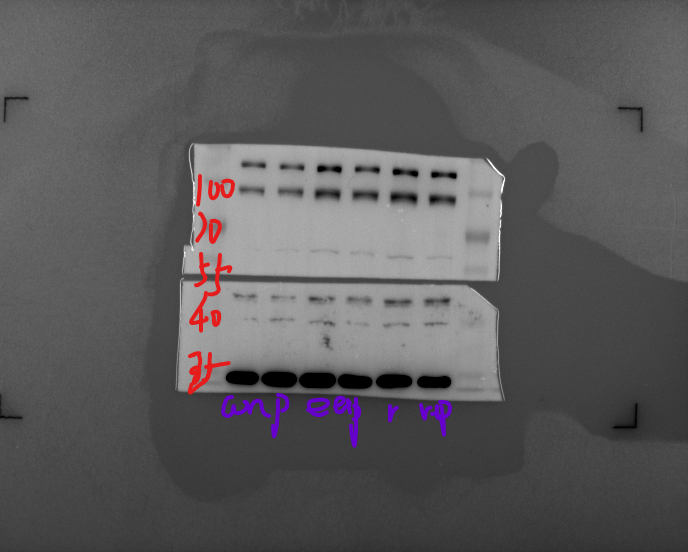


Fig 4 p53-r


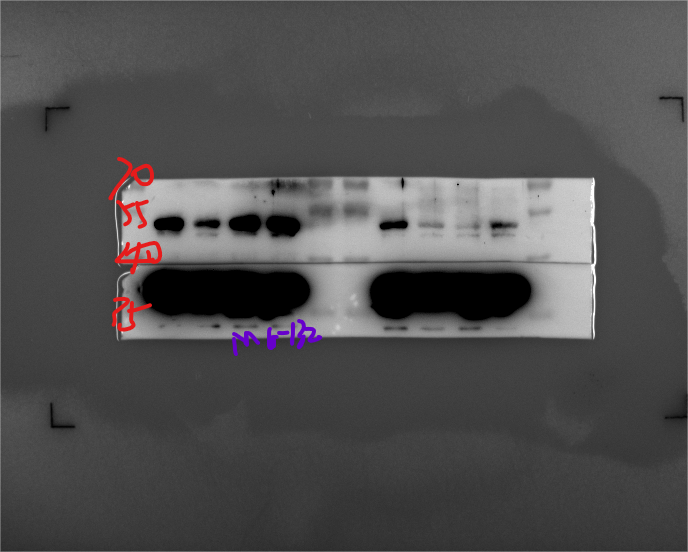


Fig 4 TFRC-gap-mg132-1


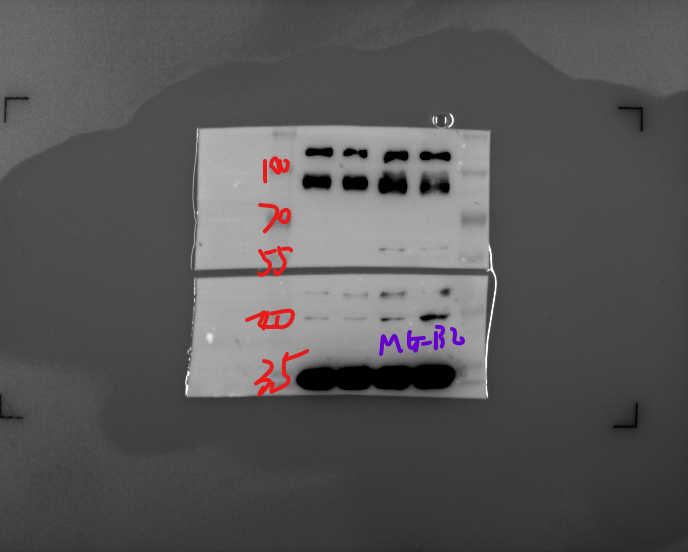


Fig 5 ACSL4


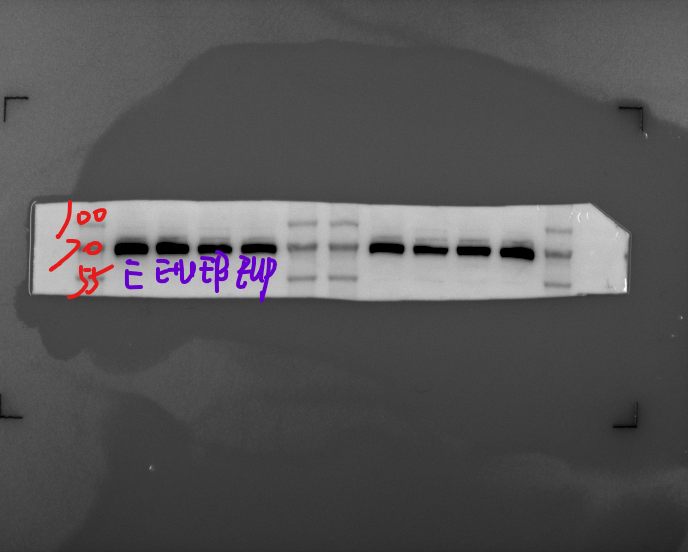


Fig 5 GPX4


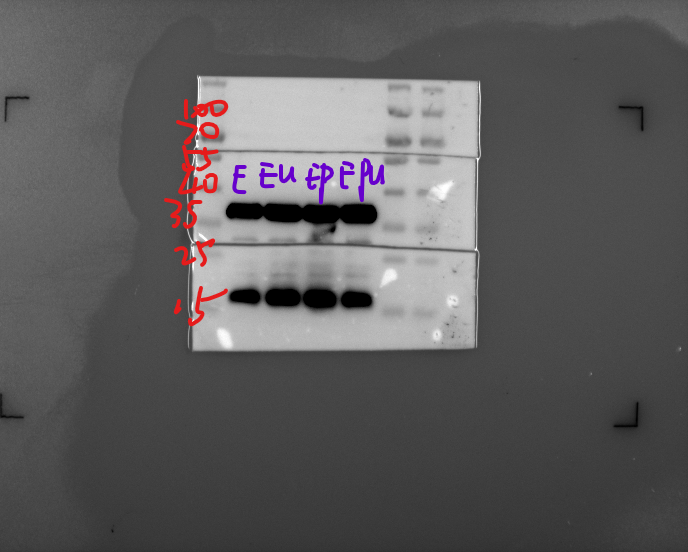


Fig 5 TfR


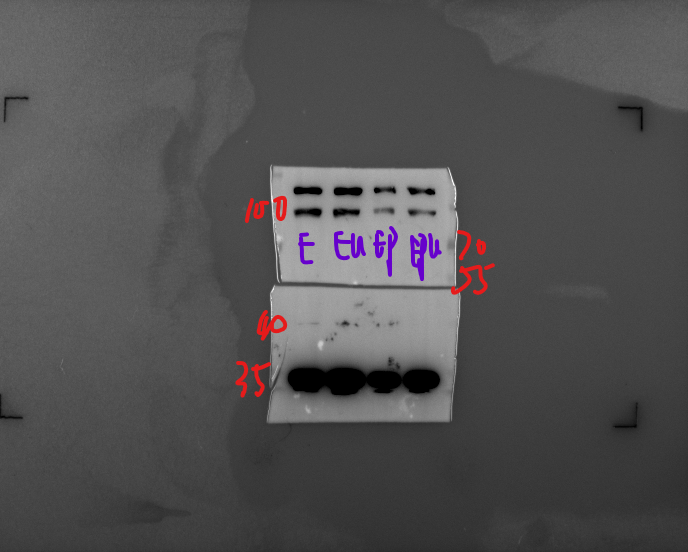


Fig 6 ACSL4


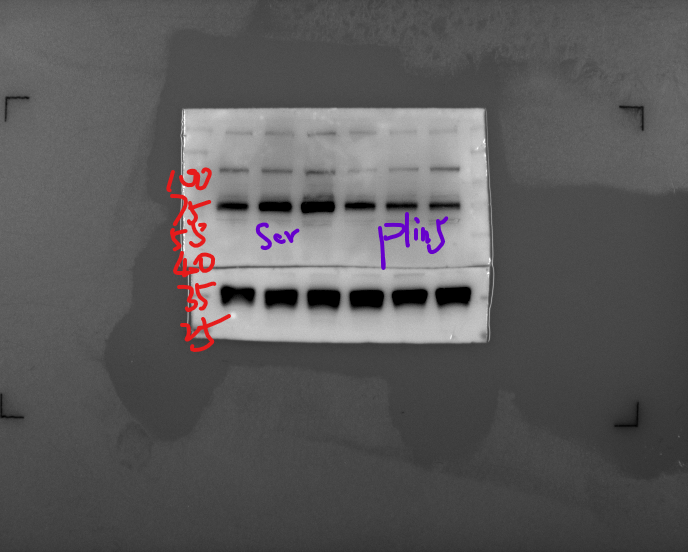


Fig 6 GPX4


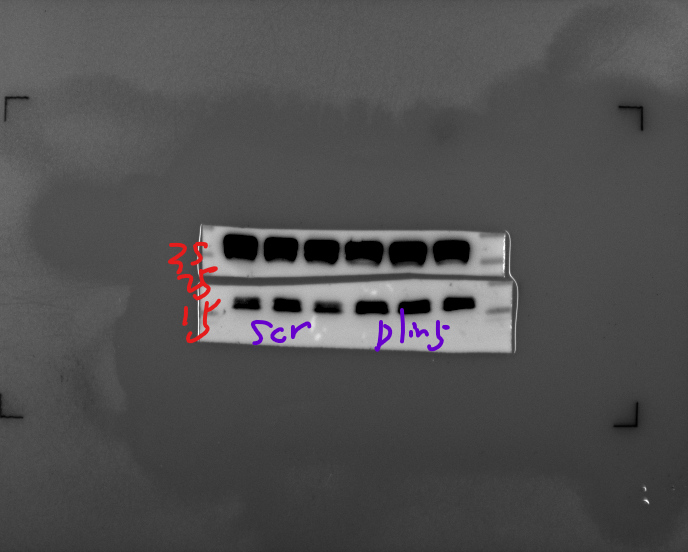


Fig 6 TFR1


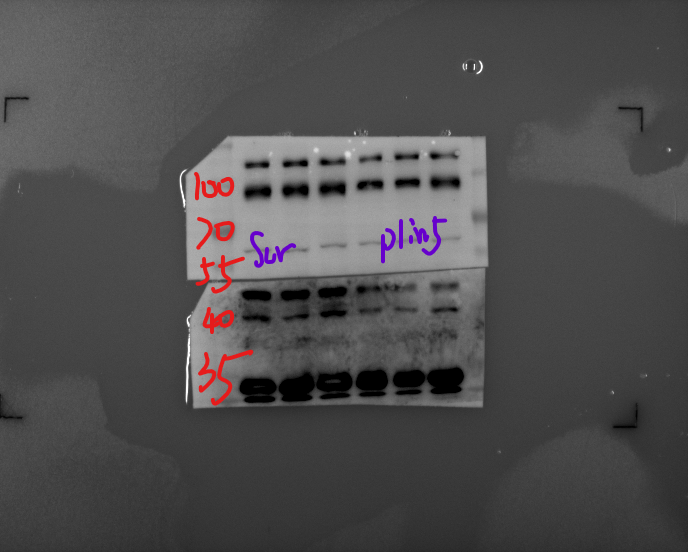

Supplement: Supplementary file 1 [file Data_Sheet_1.docx]
